# Audit: Do Electronic Mental Health Records Match General Practice Shared Records of Medications and Allergies for Patients Residing at a Community Forensic Supported Accommodation?

**DOI:** 10.1192/bjo.2022.494

**Published:** 2022-06-20

**Authors:** Kate Bernard, Neeti Sud

**Affiliations:** 1 Newcastle University, Newcastle upon Tyne, United Kingdom; 2 Cumbria, Northumberland, Tyne and Wear NHS Foundation Trust, Newcastle upon Tyne, United Kingdom

## Abstract

**Aims:**

Medicine reconciliation in community teams is guided by trust guidance, which emphasises that for all new patients accepted into a community team, staff should be aware of all current medication (both psychotropic medication and those prescribed for physical health needs). This information needs to be considered at each review to inform safe prescribing. Upon this background, concordance between electronic mental health records and general practice shared records of medications and allergy status for patients residing at a community forensic supported accommodation was audited in order to identify areas for improvement in practice.

**Methods:**

Data were collected from mental health electronic records (Rio) and general practice records (Health Information Exchange). All patients residing full-time at a community forensic supported accommodation in Cumbria Northumberland Tyne and Wear NHS Foundation Trust during January 2022 were included. Concordance between the records in medication and allergy status was assessed. Initial assessment was performed by one reviewer and 100% of included records were then cross checked by a second reviewer. Data collection was intended to pick up any mismatch in the records. Standards were set at 100% concordance.

**Results:**

Eight patients were included. For allergy status, in two patients’ (25%) records showed allergies which were present in electronic mental health records were not present in general practice records. The reasons as to lack of documentation of allergy status in general practice records were unclear. Cross check of the discharge summaries to primary care from the wards where allergies were originally identified indicates that allergies were clearly documented.

For medication, discrepancies between records were found in two patients (25%). In these patients, medications present on general practice records were not present on mental health records. These were both physical health medications (vitamin D supplements) which were being prescribed regularly by primary care and had been omitted during transcription onto electronic mental health records.

**Conclusion:**

- *1)* Currently, standard practice is for updates of medication on mental health records to take place every four months as part of quarterly care coordination reviews. Electronic mental health records should not be relied upon solely to check patients’ medication: while they provide a snapshot, cross checking with primary care records and pharmacy remains a must. This is current practice and ensures patient safety.
- *2)* Primary care to be made aware of the omissions and requested to update their records as per the discharge summaries.
- *3)* Continue regular re-audits every four months

